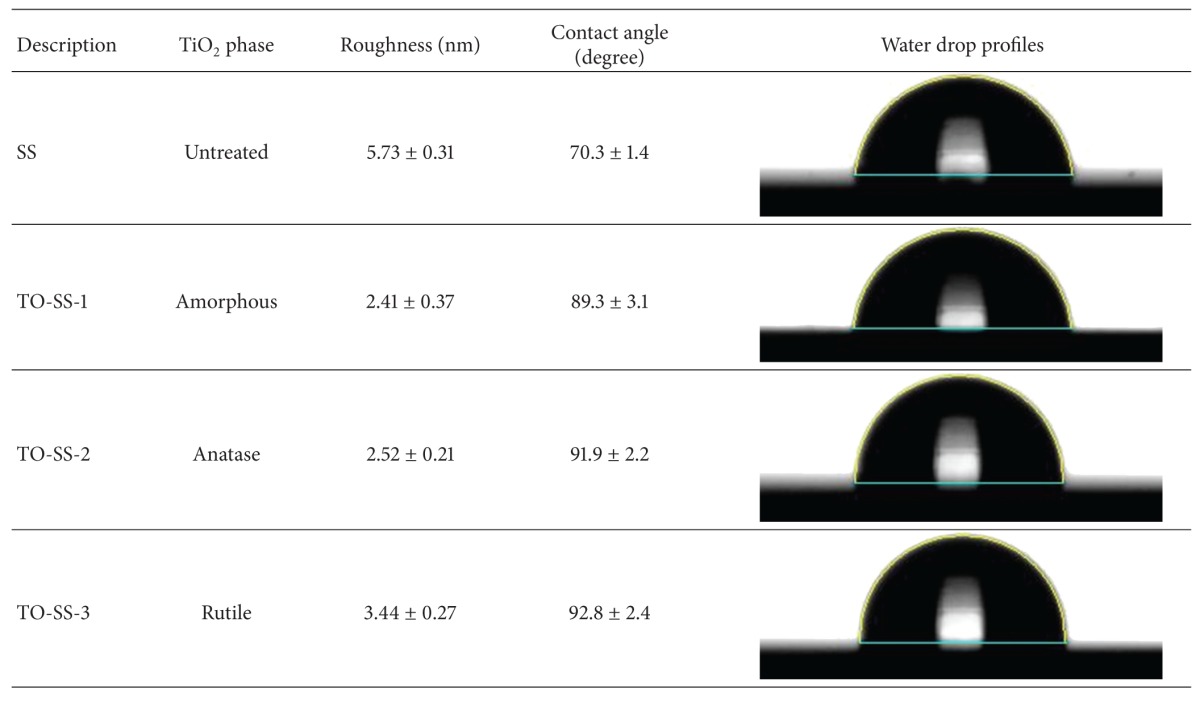# Research of Electrosurgical Ablation with Antiadhesive Functionalization on Thermal and Histopathological Effects of Brain Tissues In Vivo

**DOI:** 10.1155/2014/182657

**Published:** 2014-05-22

**Authors:** Wen-Tien Hsiao, Chun-Ming Kung, Jan-Show Chu, Keng-Liang Ou, Pei-Wen Peng

**Affiliations:** ^1^School of Dentistry, Taipei Medical University, Taipei 110, Taiwan; ^2^Department of Dentistry, Cathay General Hospital, Taipei 106, Taiwan; ^3^Department of Dentistry, Cathay General Hospital, Sijhih, Taipei 221, Taiwan; ^4^Research Center for Biomedical Devices and Prototyping Production, Taipei Medical University, Taipei 110, Taiwan; ^5^Department of Pathology, School of Medicine, Taipei Medical University, Taipei 110, Taiwan; ^6^Graduate Institute of Biomedical Materials and Tissue Engineering, Taipei Medical University, No. 250, Wu-Hsing Street, Taipei 110, Taiwan; ^7^Department of Dentistry, Taipei Medical University, Shuang Ho Hospital, Taipei 235, Taiwan; ^8^Research Center for Biomedical Implants and Microsurgery Devices, Taipei Medical University, Taipei 110, Taiwan; ^9^School of Dental Technology, Taipei Medical University, No. 250, Wu-Hsing Street, Taipei 110, Taiwan

## Abstract

Thermal injury and tissue sticking are two major concerns in the electrosurgery. In the present study, the effect of lateral thermal injury caused by different electrosurgical electrodes on wound healing was investigated. An electrosurgical unit equipped with untreated (SS) and titanium oxide layer-coated (TiO_2_-coated) stainless steel needle-type electrodes was used to create lesions on the rat brain tissue. TiO_2_ layers were produced by radiofrequency plasma and magnetron sputtering in the form of amorphous (TO-SS-1), anatase (TO-SS-2), and rutile (TO-SS-3) phase. Animals were sacrificed for evaluations at 0, 2, 7, and 28 days postoperatively. TO-SS-3 electrodes generated lower levels of sticking tissue, and the thermographs showed that the recorded highest temperature in brain tissue from the TO-SS-3 electrode was significantly lower than in the SS electrode. The total injury area of brain tissue caused by TO-SS-1 and TO-SS-3 electrodes was significantly lower than that caused by SS electrodes at each time point. The results of the present study reveal that the plating of electrodes with a TiO_2_ film with rutile phases is an efficient method for improving the performance of electrosurgical units and should benefit wound healing.

## 1. Introduction

Electrosurgery has been widely accepted by physicians to aid in the removal of tumors since its original application by Bovie and Cushing in the 1920s. The basic components of a monopolar electrosurgery consist of the high-intensity radiofrequency (RF) generator, the active electrode, the dispersive pad, and the patient. RF alternating current flows through tissue from an active electrode to a dispersive pad, resulting in tissue cutting, coagulation, and ablation [1].

RF tissue ablation makes thermal lesions and tissue coagulation around the tip of an active needle electrode. Although needle ablation has been widely applied to cardiology, urology, neurosurgery, and otolaryngology, there are still rare but occasionally serious complications that accompany the procedure [2]. The heat can destroy the targeted tumor in a range of 65–75°C, and the temperatures above 75°C cause significant adjacent structures destruction [3]. Nontarget thermal damage to vital structures and tissue destruction adjacent to targeted lesions are the common complications from the needle ablation due to a rapid local temperature rise from the high current density. Carbonized charred tissue sticking to the electrode causes an abrupt drop in current density and eventually ceasing the operation [4]. Meanwhile, the sufficient lesion depth and diameter are difficult to obtain. To address these concerns, the RF ablation has incorporated different strategies to minimize the lateral spread of thermal energy and carbonized charred tissues, such as the automatic thermal monitoring system [5], the image-guide system [6], and the percutaneous saline-enhanced and impedance-controlled system [7]. Recently, a relative simple method, the surface modification of the electrode, has been introduced to prevent lateral thermal damage and increase the precision of the lesion depth and diameter. Çeviker et al. [8] introduced the Teflon- (tetrafluoroethylene, PTFE-) coated electrode substrate to reduce the level of tissue sticking and charring. Simmons et al. [9] compared the gold- and platinum-coated electrodes on myocardial lesion size and suggested that deeper lesions should be able to be made when RF energy is delivered to a gold rather than platinum tip electrode. However, the optimal approach is still under development and an alternative coating material, titanium dioxide (TiO_2_), has attempted to overcome the clinical drawbacks in the present study.

TiO_2_ was used in a wide range of technological applications due to its high refractive index, excellent transmittance, and photocatalytic property [10, 11]. Recently, TiO_2_ has been reported to be good biocompatible and blood compatible. The TiO_2_ group is composed of three types of crustal structures, and especially the rutile and anatase phases have evolved as the model system in the surface coatings for biomedical materials [12]. Rutile and anatase phases have similar band-gap energies and they can be changed from insulator to a semiconductor by means of altering processing parameters [13].

The present study was directed toward quantification and comparison of the lesion position and thermal distribution produced by the monopolar RF energy application with different coatings. Specifically, the present study evaluated the effect of the various coatings of monopolar on the short-term histologic properties of brain tissue in an in vivo rat model.

## 2. Materials and Methods

### 2.1. TiO_2_ Thin Film Deposition and Property Evaluations

A deposition process that combined radiofrequency plasma and magnetron sputtering system equipped with one pure Ti target (99.99% purity) was utilized to deposit the TiO_2_ film on commercial electrode (needle-type austenite AISI 304 stainless steel denoted as a SS electrode). All of the substrates were cleaned in an ultrasonic bath with a sequence of acetone and ethanol for 15 min followed by air drying before being loaded into the chamber. Subsequently, the chamber was evacuated at vacuum pressure 2.0 × 10^−6^ torr for 15 min and then the substrates were presputtered by Ar^+^ cleaning for 10 min to remove the native adsorbed contaminants and impurities under a radiofrequency power 225 W with chamber running pressure 8.0 × 10^−3^ torr. After cleaning process, a mixture of O_2_/Ar with a fixed flow ratio (40/60) was introduced through mass flow controller to keep a working pressure of 8.0 × 10^−3^ torr. Then, the substrate holder was heated and kept at 300°C during the following deposition. The untreated Ti was treated at a negative bias voltage of 200 V and the varying powers for 30 min, creating a controlled TiO_2_ layer. For ease of identification, the SS electrodes coated with TiO_2_ layer at the amorphous, anatase, and rutile phases were labeled as TO-SS-1, TO-SS-2, and TO-SS-3 electrodes, respectively.

Raman spectra were recorded using the Horiba HR800 (Protrustech Co., Ltd., Taipei, Taiwan) with a 633 nm laser to detect the TiO_2_ phases. Topographical analyses were conducted using an atomic force microscope (AFM, DPN 5000, NanoInk, Skokie, IL, USA). The silicon nitride probe was scanned over a 1 *μ*m × 1 *μ*m area in the tapping mode. The contact angles of the specimens were assessed by dropping 0.4 mL of distilled water on specimens and measured using a video-based goniometer (EA-01, Jeteazy Co., Ltd., Hsinchu, Taiwan). The specimens were tested 6 times to obtain average contact angle values.

### 2.2. In Vivo Study

#### 2.2.1. Animal Models

RF-induced thermal lesion experiments were conducted in normal rat brain model. The animal procedures were approved and conducted by the Institutional Animal Care and Use Committee at Taipei Medical University (number LAC-99-0037). A total of 48 male Sprague-Dawley rats (weighing 276–300 g, BioLASCO Taiwan Co., Ltd., Taipei, Taiwan) were purchased and housed in cages for 14 days prior to experimentation. Food and water were available ad libitum. The animals were randomly allocated into 3 different experimental groups of 16 animals each.

#### 2.2.2. Surgical Procedures

Aseptic precautions were utilized in all surgical procedures. The animals were positioned in a stereotactic frame under pentobarbital anaesthesia (40 mg/kg IP). Bilateral cranial burr holes (1 mm) were drilled on the right and the left, at the coronal suture 4.0 mm lateral to the midline. Lesions were performed bilaterally using an ERBE ICC 300 RF generator (Elektromedizin GmbH, Tübingen, Germany). A monopolar current at 20 W power setting was used and the duration of each penetration was 3 s. Three TiO_2_-coated electrodes (TO-SS-1, TO-SS-2, and TO-SS-3) were evaluated and the regular SS electrode without any coatings was used as the control (SS). The TiO_2_-coated electrode was applied to one side and the SS electrode to the other. Performance of each electrode group was determined based on thermometry, tissue sticking, and histological examination.

#### 2.2.3. Real-Time Thermometry and Quantification of Tissue Sticking

Real-time thermometry data was recorded from the initiation to completion of each lesion using a thermal-imaging infrared camera with the thermal analysis simulation software (Advanced Thermo TVS-500EX, NEC Avio Technologies, Tokyo, Japan). The weight of each electrode was measured before and after the operation. The degree of tissue sticking was calculated as the weight of sample after operation minus the weight of sample before operation.

#### 2.2.4. Injury Area and Histological Examination

Rats in each group were sacrificed after 0, 2, 7, and 28 days' recovery period (*n* = 4). Perfusion was done with 0.9% saline and then paraformaldehyde. Brains were taken and fixed in 10% buffered formalin and the samples covering lesions were cut from the brain specimens. They were treated in a graded alcohol series and embedded in paraffin. Continuous tissue sections (3 *μ*m) for all brain samples were taken for hematoxylin and eosin (H&E, 3008-1&3204-2, Muto, Japan). Stained samples were observed under a light microscope (BX51, Olympus, Japan). The total injury area in lesions for each group was quantified by the image software (SPOT basic software, SPOT imaging solutions, MI, USA).

### 2.3. Statistical Analysis

Statistical analyses were performed using the commercially available software program, SPSS 14.0 (Statistical Package for the Social Sciences, SPSS Inc., Chicago, IL, USA). For each experiment, data from 6 replicates were expressed as mean ± standard deviation (SD) per variable, which were repeated to ensure validity. Analysis of variance (ANOVA) for comparison between groups was first performed and then Student-Newman-Keuls test was used in every other group. The statistical significance level was set at *P* < 0.05.

## 3. Result and Discussions

**Figure 1:**
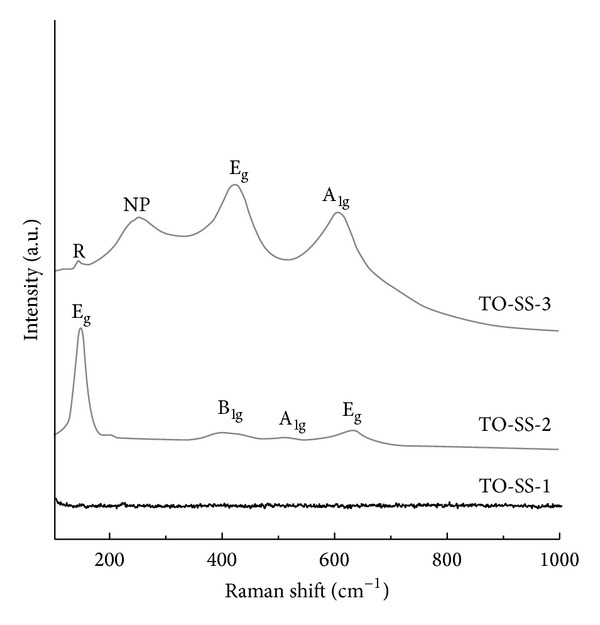
Raman spectrum of SS and TiO_2_-coated electrodes.

Raman spectra of TiO_2_-coated electrodes are shown in Figure 1. No peaks were observed in the Raman spectra of the TO-SS-1 electrode. For the TO-SS-2 electrode, the Raman lines at 151, 409, 515, and 633 cm^−1^ were assigned as the E_g_, B_1g_, A_1g_ or B_1g_, and E_g_ modes of the anatase phase, respectively, indicating that an anatase phase was formed in the SS electrode [14]. For the TO-SS-3 electrode, a band at 142.3 cm^−1^ was related to the long-range ordered structure of the crystalline rutile phase [15]. The 245.2 cm^−1^ mode is frequently observed in nanophase TiO_2_, whereas the mode at 442.2 is associated with the E_g_ phase of the rutile phase, and 609.5 cm^−1^ is associated with the A_1g_ modes of the rutile phase [16].

**Figure 2:**
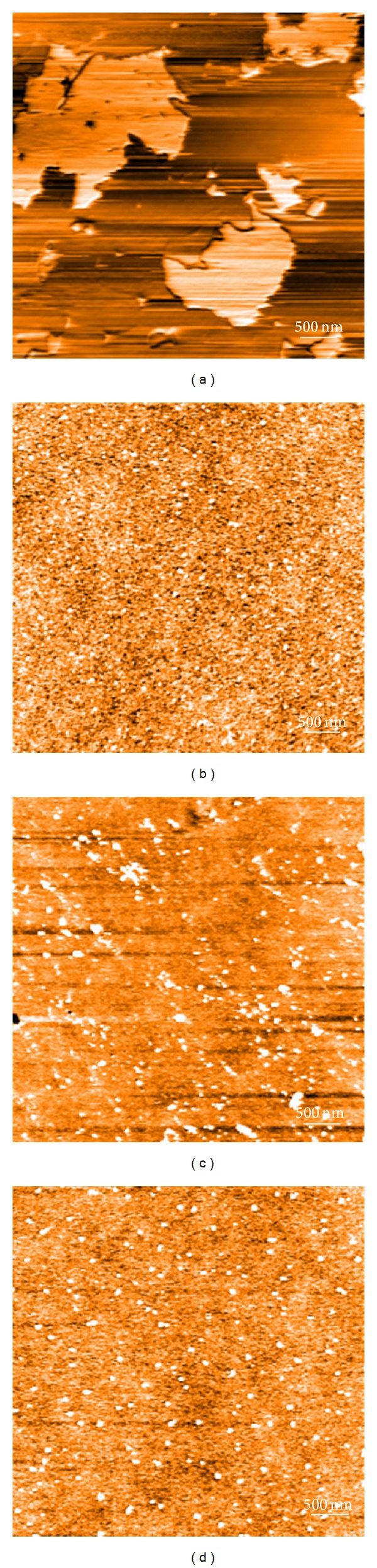
AFM tapping mode images (scan area 5 *μ*m × 5 *μ*m) of (a) SS, (b) TO-SS-1, (c) TO-SS-2, and (d) TO-SS-3 electrodes ((a, c) are 2D mode and (b, d) are 3D mode).

Figure 2 shows the surface morphologies of the SS and TiO_2_-coated electrodes using an AFM, which were acquired in a scanned range of 5 × 5 *μ*m^2^. The SS electrode exhibited relatively planar surfaces with parallel polishing traces, as shown in Figure 2(a). The TiO_2_-coated electrodes exhibited similar topographies as shown in Figures 2(b) to 2(d), which exhibited the formation of a more uniform and denser TiO_2_ layer nanostructure with round grains.  Table 1 lists the average surface roughness values. There was no difference between groups.

The contact angle of distilled water on SS and TiO_2_-coated surfaces was examined, with results shown in Table 1. The SS surfaces had the lowest value (70.3 ± 1.4), whereas TO-SS-1 surfaces exhibited comparative hydrophobicity, with a value of 92.8 ± 2.4. One-way ANOVA revealed a significant difference between the SS and TiO_2_-coated electrodes (*P* < 0.05). However, no significant difference between the TiO_2_-coated electrodes was observed (*P* > 0.05).

**Figure 3:**
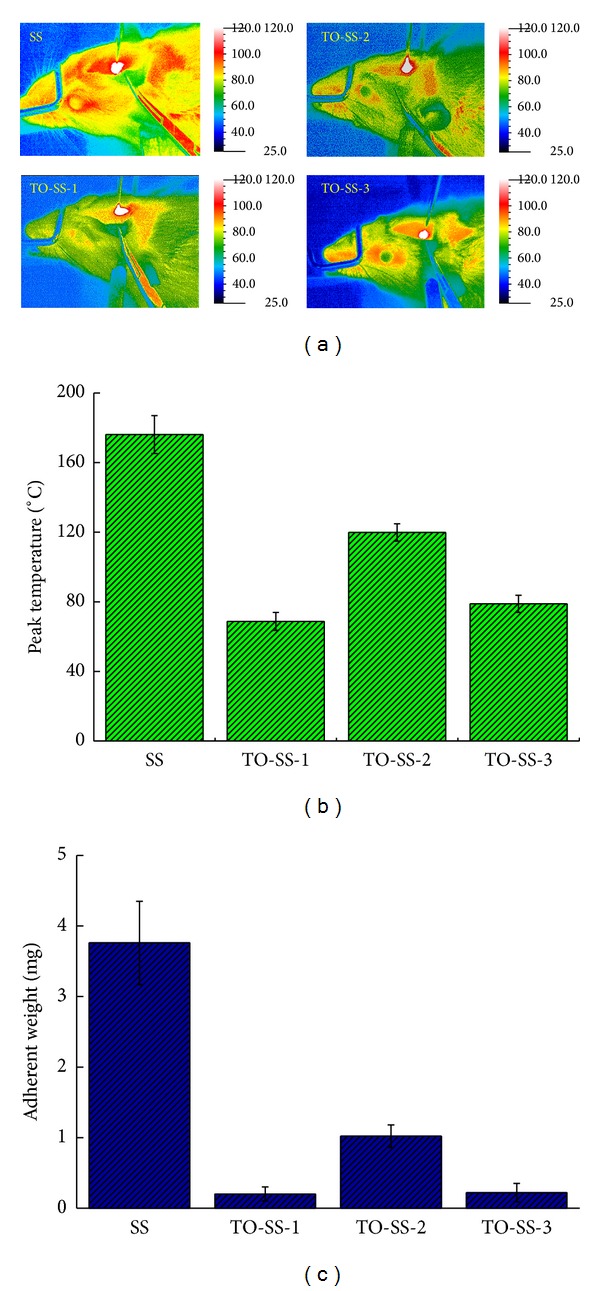
Comparisons of (a) thermographs, (b) the peak temperature, and (c) adherent weight for SS and TiO_2_-coated electrodes.

The temperature distributions in ex vivo rat brain tissue around the SS and TiO_2_-coated electrodes measured using an infrared thermal imaging camera are shown in Figure 3(a). A similar temperature distribution was found in experiments carried out with each needle type. Carbonization and tissue sticking occurred at the needle tips of each electrode, where the maximum temperatures were found in a concentric ring 3–5 mm. The highest temperature recorded while applying a SS sample was 176.18 ± 10.87°C. The highest temperatures recorded for TO-SS-1, TO-SS-2, and TO-SS-3 electrodes were 68.73 ± 5.12°C, 119.85 ± 5.01°C, and 78.83 ± 4.92°C, respectively, which were significantly lower than that in the SS group (*P* < 0.01) (Figure 3(b)). Electrodes were weighed before and after the operation for each group in order to measure the amount of tissue sticking to the needle tip (Figure 3(c)). The SS electrode needle tip showed a significantly higher amount of adhering tissue than the TiO_2_-coated electrode (*P* < 0.05). Among the TiO_2_-coated electrodes, the TO-SS-2 electrode exhibited the lowest amount of tissue adherent.

**Figure 4:**
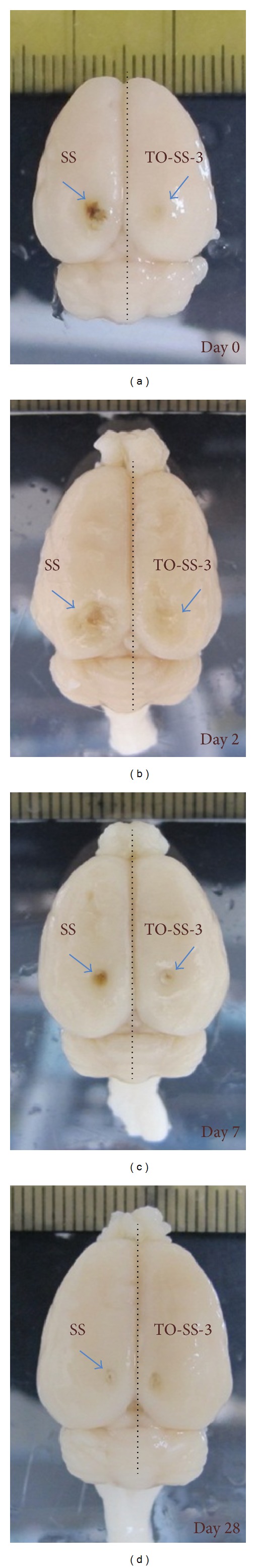
Gross observations showed the injury site created by SS (left) and TO-SS-3 (right) electrodes at (a) day 0, (b) day 2, (c) day 7, and (d) day 28.

Pictures shown in Figure 4 reveal the difference in the total lesion areas caused by SS and TO-SS-3 electrodes. The injury created by the SS electrode was larger than that of the TO-SS-3 electrode as shown in Figure 4(a). The borders of the lesions sites for both electrodes were beginning to heal by the end of day 2, being both wider and shallower than immediately after the operation (Figure 4(b)). By day 7 the lesion areas were smaller for both the SS and TO-SS-3 electrodes, and few blood cells remained (Figure 4(c)). By the end of day 28, the lesion created by TO-SS-3 electrodes was healed, and the small lesion site created by SS electrodes still remained as shown in Figure 4(d).

**Figure 5:**
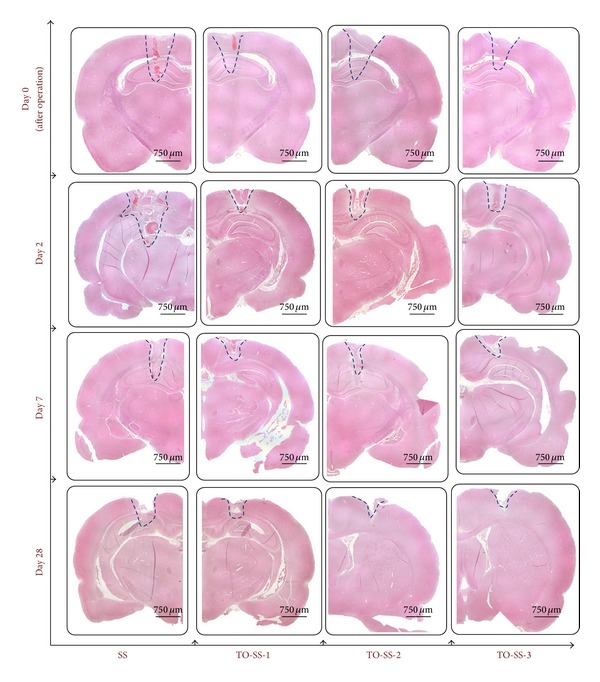
Histological observation of injury area caused by SS and TiO_2_-coated electrodes at the end of day 0, day 2, day 7, and day 28.

Histological examination of specimens for all electrode types showed varying amounts of coagulation necrosis and bleeding, indicating heat damage to the tissue as shown in Figure 5. When TO-SS-3 and TO-SS-1 electrodes were used, no obvious bleeding was detected postoperatively in treated tissues. In contrast, hemorrhaging caused by SS and TO-SS-2 electrodes was evident. At day 2, hemorrhaging in SS groups was markedly higher than in the other groups, and infections and apoptosis were observed at the treated site. The thermal-injury areas caused by SS electrodes were larger than those by the TiO_2_-treated electrodes, with the least tissue damage found when using the TO-SS-3 electrode. The thermal-injury areas tended to diminish for all groups at the end of day 7.

Commercially available electrosurgical electrodes are usually coated with Teflon to avoid tissue sticking. However, surgical smoke with an unfavorable flavor encouraged the advanced approaches. The objective of the present study was to establish an in vivo rat model to study the thermal effects on damage to brain tissue caused by electrosurgical ablation with TiO_2_-coated needle-type electrodes.

It is well known that titanium possesses its excellent biocompatibility due to its very stable and corrosion resistant oxide layer [17, 18]. TiO_2_ layers have been considered a promising coating for implants with proven biocompatibility and blood compatibility [13, 19]. Therefore, coatings of TiO_2_ layers have been deposited on various devices via various technologies to increase clinical effectiveness. By means of radiofrequency plasma and magnetron sputtering, a layer of rutile, anatase, or amorphous phase TiO_2_ layer was deposited on commercially available electrodes.

Tissue thermographs showed that temperatures recorded for TO-SS-3 electrodes were significantly lower than those for SS electrodes under the same RF power setting. This can be attributed to the high electric and thermal conductivity of rutile phase TiO_2_ film [20]. Hence a TO-SS-3 electrode can deliver electrical energy to the target tissue more efficiently. This can prevent overheating of the electrode substrate and reduce the incidence of thermal injury. Macroscopic observation and histological examination showed clearly that TO-SS-3 electrodes produce a smaller area of injury than commercial SS electrodes.

## 4. Conclusion

A TiO_2_ film accompanied with hydrophobic surface can be formed and bonded on the needle-type electrode substrate via radiofrequency plasma and magnetron sputtering system. The enhanced thermal conductivity and surface hydrophobicity of TiO_2_ coatings with rutile phase can improve the performance of electrosurgical electrodes, in terms of tissue sticking and thermal injury. TO-SS-3 electrodes had lower levels of sticking tissue with lower surgical temperatures during electrosurgery. The total injury area of rats brain tissue treated with TO-SS-3 electrodes was significantly smaller than those of rat brain tissues treated with SS electrodes at all time points. This study reveals that the plating of TiO_2_ coatings on electrode substrates is a simple and effective means of improving the performance of electrosurgical units.

## Figures and Tables

**Table 1:** Surface roughness, contact angle, and water drop profiles for SS and TiO_2_-coated electrodes.